# Medical staff’s sense of awareness of informed consent for adolescent cancer patients and the need for decision-making support practiced from the perspective of trauma-informed approach

**DOI:** 10.1186/s12910-023-00907-y

**Published:** 2023-05-06

**Authors:** Kyoko Tanaka, Maoko Hayakawa, Makiko Mori, Naoko Maeda, Masako Nagata, Keizo Horibe

**Affiliations:** 1 National Center for Child Health and Development, Setagaya-ku, Tokyo, Japan; 2 Ochanomizu University Human Developmental Sciences, Bunkyo-ku, Tokyo, Japan; 3 Department of Hematology/Oncology, Saitama Children’s Medical Center, Saitama, Saitama Japan; 4 Department of Pediatrics, National Hospital Organization Nagoya Medical Center, Nagoya, Aichi Japan; 5 Psychological Support&Research Center for Human Development, NAGOYA UNIVERSITY, Nagoya-shi, Aichi Japan; 6 Division of Consultation liaison Department of Psychosocial Medicine, 2-10-1 Okura, Setagaya- ku, Tokyo, 157-8535 Japan

**Keywords:** Decision-making, Decision-making capacity, Adolescent, Informed consent, Trauma-imformed

## Abstract

It has not been established how to assess children’s and adolescents’ decision-making capacity (DMC) and there has been little discussion on the way their decision-making (DM). The purpose of this study was to examine actual situation and factors related to difficulties in explaining their disease to adolescent cancer patients or obtaining informed consent (IC). The cross-sectional questionnaire survey was conducted. Physicians who have been treating adolescent cancer patients for at least five years answered a self-administered questionnaire uniquely developed about clinical difficulties in explaining, IC and factors related patient’s refusal of medical treatment (RMT). Descriptive statistics for each item and a polychoric correlation analysis of the problems and factors related to the explanation were conducted. As a result, fifty-six physicians were participated (rate of return: 39%). Explaining the disease and treatment to patients (83.9%), IC to patients (80.4%), and explaining the disease and treatment to parents (78.6%) was particularly problematic. Difficulties to provide support related with patient’s refusal of medical treatment and to explain disease and treatment for patient and parents were related to difficulties obtaining IC for the patient. *Conclusion*: There are clinically difficult to explain for the patient or parents and to obtain IC for the patient. It is necessary to establish a disease acceptance assessment tool for the adolescence generation so that it can be applied in the field.

**Supplementary Information:**

The online version contains supplementary material available at 10.1186/s12910-023-00907-y.

## What is known

- Decision-making for adolescent patients needs to take into account the influence of others because they are in the process of decision-making capacity development and establishing independence and self-identity.
- It is not clear what physicians think about that.

## What is new

- Our study revealed the physicians feel difficulties to provide support related with patient’s refusal of medical treatment and to explain disease and treatment for patient and parents were related to difficulties obtaining IC for the patient.
- The outcome suggests the needs to develop assessment tools that evaluate the psychosocial factors behind patients’ refusal and the needs of patient and their families.

## Introduction

It is an ethical imperative in medicine that the decision-making process should proceed after the patient has been properly informed of the name, condition, and treatment of the disease. The importance of principles of the right to self-determination is no different in child and adolescent patients [1–3]. It is an important issue in pediatric medicine for medical provider obtain informed consent (IC) from the patient as adherence to the principle of the right to self-determination and children’s rights. However, the assessment of child and adolescent’s decision-making capacity (DMC) and the best way to support decision-making (DM) has not yet been established and urgently need to be discussed.

It has been suggested that illness experience, family situation, family structure, and culture all influence children’s DMC [4]. Furthermore, DMC is influenced not only by parents, but also by physicians because of their medical dependance and by friends because of their developmental significance [5].

With regard to the age at which a child’s ability to consent to medical care is recognized, it is reported that in the United Kingdom it is 16 years of age or older for minors, in Denmark it is 15 years of age or older, and in the United States it varies by state. In Japan, however, there is no such law, but the ability to consent is recognized at 15 years of age or older for cadaveric organ transplants and at 18 years of age or older for wills. Against this background, the legal and medical communities have had multiple discussions on how to incorporate and practice decision-making in Pediatrics in Japan, which has ratified the Convention on the Rights of the Child. Based on this background, the author and our colleagues have taken the lead in formulating the Children’s Charter for Medical Care at the Japan Pediatric Society in 2022 [6]. In particular, the Charter states that children’s opinions are to be expressed in an age-appropriate manner, that explanations are to be provided in a manner that is easy to understand according to the child’s development, and that children’s questions are to be properly answered by medical staff to gain the child’s understanding.

In recent years, the field of cancer care has been focusing on the life stages of patients, aiming to establish a system of medical care coordination that takes into account the disease composition, individual needs, and lifestyle, and to promote measures to encourage patients’ independence and self-management. Although it has been argued that the support for adolescence generation patients including 10 to 24 years old [7] is needed, those needs are unmet. In particular, IC for adolescent patients needs to take into account the influence of others in the assessment of DMC because the adolescent cancer patients are in the process of DMC development and are affected by the presence and opinions of others in the process of establishing independence and self-identity.

The adolescent generation is a stage of life in which they are in the process of acquiring self-identity and have a unique adolescent mentality. Undergoing cancer treatment during this time has a significant impact on adolescents, not only physically, but also psychosocially [8, 9].

First, there is the psychological impact of cancer treatment itself. Cancer treatment is characterized by treatments with severe side effects; treatments with severe pain, such as intrathecal injections; changes in appearance and functioning due to chemotherapy, steroids, and surgery; limitations due to long and frequent hospitalizations and sterile room management; and repeated highly invasive treatments and tests. In pediatric medicine, the psychological negative reactions of patients and families related those medical process, referred to as pediatric medical traumatic stress (PMTS) [10, 11] or the concept of medical trauma.

And both these emotional reactions and medical trauma are known to significantly impair psychosocial functioning in adolescents and influence their decisions about various medical procedures [12]. In other words, it may be important to engage in trauma-informed responses to psychological stress reactions related to illness and treatment in order to support active, more autonomous decision-making among adolescents.

Moreover, participation in treatment decisions is especially important for adolescents, for whom independence and autonomy are developmental issues. In the midst of many experiences of coercion and dependence, adolescents’ exercise of autonomy is likely to lead to autonomy and self-efficacy.

Therefore, this study was conducted to understand the actual situation of explaining the disease condition and obtaining IC for adolescent cancer patients in Japan, and to examine the factors related to the confusion of explaining the disease condition, obtaining IC from them, the refusal of medical treatment, and the decision-making process.

## Methods

### Survey participants

Physicians who belong to a pediatric cancer center hospital in Japan with at least five years of clinical experience in treating child and adolescent generation cancer patients.

### Survey contents

We conducted a self-administered questionnaire survey. The questionnaire was developed by the first author based on questions used in two previous surveys of pediatricians who treat patients with chronic pediatric diseases and the results of their responses, as well as modifications made in response to their responses, and with the addition of specific experience in pediatric oncology. Pediatric hematology/oncology specialists, pediatricians, and clinical psychologists reviewed the questionnaire items. Finally, the questionnaire consisted of 40 items in five domains asking about clinical difficulties, policies, responses, and support in clinical practice regarding explanation and consent to patients. The domains, each number of items and response methods are shown in Table 1. Adolescent generation in this study is defined as 12 to 20 years old.

**Table 1:** Survey items and each response methods

Domain	Item	Response method
1. Clinical difficulties about consent and assent	[1] patients’ resistance to and refusal of medical treatment (RMT), [2] assessment of and response to patients’ RMT, [3] support for patients’ RMT, [4] informed consent for patients, [5] explanation of illness and treatment for patients, [6] assessment of and response to parents’ RMT, [7] explanation of illness and treatment for parents, and [8] others.	For items [1] through [7], we asked for responses on a five-point scale of “often”, “sometimes”, “neither”, “rarely”, and “never”. Item [8] was an open-ended question.
2. Explanation for the patient	Frequency of patient explanations in medical treatment, target age group, contents of explanations, target conditions, importance of explanations, decision-makers for implementation of explanations, reasons for not providing explanations, main explainers, and methods.	Multiple-choice
3. Patient’s RMT	Factors that may be related to patients’ and parents’ refusal of medical treatment and parents’ refusal to obtain IC/IA.	Multiple-choice
4. Important factors for patient explanation, obtaining consent/assent, and respecting wishes	Six factors related to obtaining IC and IA, such as the patient’s own right to make decisions and the need to respect the patient’s own will and opinion. Age and importance of following the patient’s intentions.	Multiple choice If “Other” was selected was free description. Item of age was free description.
5. Assessment of the patient’s understanding	Whether or not an assessment of patient understanding was conducted, who conducted it, and how it was conducted.	Multiple-choice If “Other” was selected was free description. Item of “Detail and Contents” was free description.

RMT, refusal medical treatment

### Survey methods

The same number of questionnaires and reply envelopes as the number of physicians affiliated with the center were distributed to center directors of 15 pediatric cancer centers designated by the Minister of Health, Labor and Welfare as central facilities providing pediatric cancer care and support in the community. They distributed the survey forms to physicians who met the eligibility criteria. An explanatory note clearly stating the purpose of the survey was attached to the questionnaire, and the reply to the questionnaire was deemed to be consent for research cooperation. The survey period was from January to May 2020.

### Data analysis

First, descriptive statistics for each item were compiled. Next, we conducted a polychoric correlation analysis of the problems in clinical practice regarding explanation and consent, and examined the factors related to the explanation of disease status for adolescent cancer patients. Statistical significance was analyzed by using EZR [13], which is a graphical user interface for R The R version was 4.0.5 (2021-07-06) [14].

### Ethical considerations

The survey was approved by the Ethics Committee of the National Center for Child Health and Development (2019-084).

The questionnaire used for this study is shown in the supplement file.

## Results

The number of copies distributed was 143, and 56 participants completed and returned the questionnaire. The response rate was 39.2%.

### Descriptive statistics

#### Clinical difficulties about consent and assent (Table 2)

**Table 2:** Descriptive statistics of clinical difficulties regarding consent and assent

Items	Counts (% of total, N = 56)				
	Often	Sometimes	Neither	Rarely	Never
1. Patient’s RMT	1 (1.8)	24 (42.9)	2 (3.6)	28 (50.0)	1 (1.8)
2. Assessment and dealing with parent’s RMT	3 (5.4)	24 (42.9)	4 (7.1)	24 (42.9)	1 (1.8)
3. Support of patient’s RMT	7 (13.0)	24 (44.4)	3 (5.6)	18 (33.3)	2 (3.7)
4. IC to patients	28 (50.0)	17 (30.4)	5 (8.9)	6 (10.7)	0 (0.0)
5. Explanation of disease and treatment to patients	26 (46.4)	21 (37.5)	3 (5.4)	6 (10.7)	0 (0.0)
6. Assessment and dealing with parent’s RMT	3 (5.4)	20 (35.7)	4 (7.1)	25 (44.6)	4 (7.1)
7. Explanation of disease and treatment to parents	30 (53.6)	14 (25.0)	2 (3.6)	7 (12.5)	3 (5.4)

IC, informed consent; RMT, resistance to or refusal of medical treatment.

When the participants were asked about the clinical difficulties regarding consent and assent, the top three items responded as “often” or “sometimes” were the following: “explanation of disease and treatment for patients” (83.9%), “IC for patients” (80.4%), and “explanation of disease and treatment for parents” (78.6%).

#### Explanation for the patient

Over three-fourths of the participants (76.8%) reported that the explanation of medical treatment was implemented for all cases, 19.6% reported it was implemented in some case, and 8.9% reported it depended on the contents. In terms of the explanation for patients, the name of the diagnosis, the condition and pathology of the disease, the treatment and its methods, treatment and its meaning, and treatment and its side effects, over 89.3% of participants reported that the explanation was implemented in principle. On the other hand, it was reported in 71.4% of participants that in principle they would explain late complications, fertility, schooling/employment, and support available to patients. When participants were asked about the explanation of the condition and pathology of the disease, in the case of relatively minor injuries or not immediately life-threatening, the implementation rate of explanation of the condition exceeded 94.6%, whereas in the case of a serious life-threatening condition, the rate decreased to 73.2%, and in case where recovery through cure was not expected, the rate was 26.8%. Explanations were mainly given by physicians, and 19.6% of the participants answered that explanations were given on a case-by-case basis. As for the way of patient explanation, interventions using pictures and charts were often taken (94.6%). As for the decision on whether or not to provide patient explanations, the most common answer was “at the discretion of the multidisciplinary team” (55.4%) and “with the patient’s wish” accounting for only 17.9%.

The top reasons why they did not explain for patients were as follows: “it causes anxiety to patients” (67.1%) and “the patient not able to understand” (51.8%). The top reason for not obtaining consent/assent was “because patient does not have the capacity to make decisions” (60.7% consent, 64.3% assent).

In terms of whether to perform medical treatment when they did not obtain patient’s consent, when the guardians refused, for 5.4% of participants treatment was performed in principle, but when the guardian’s consent was obtained, for 41.1% of the participants treatment was performed.

#### Patient’s RMT (Table 3)

“Physical pain and suffering caused by treatment” was the most common reason for RMT among both patients and guardians (75% for patients, 51.8% for guardians).

**Table 3:** Factors related to patient’s RMT

Contents	Counts (% of total)					
	Patient		Guardian medical procedure		Guardian patient’s IC	
N	56	100	55	100	56	100
Physical pain and suffering from treatment	42	75.0	29	51.8	-	-
Emotional instability	27	48.2	11	19.6	20	35.7
Patients not being informed about the disease	18	32.1	8	14.3	20	35.7
Intentions of parents or guardians	8	14.3	-	-	-	-
Patients not being informed about treatment and procedures	16	28.6	-	-	-	-
Emotional instability of parents	-	-	31	55.4	30	53.6
Intentions of the patient	-	-	5	8.9	2	3.6
Parents’ lack of knowledge and understanding of treatment and procedures	-	-	33	58.9	22	39.3
Other	1	1.8	3	5.4	3	5.4

IA, informed assent; IC, informed consent; RMT, resistance to or refusal of medical treatment.

#### Important factors for patient explanation, obtaining consent, and respecting wishes (Table 4)

In all items, the highest response was given to “patient’s capacity to understand” (75% explanation, 78.6% consent, 33.9% respect for wishes). When there is a guideline regarding age for the relevant action, explanation for patients and obtaining their assent were performed at an average age of 8 years, obtaining their own consent at an average age of 12.7 years, and respecting their own wishes to RMT at an average age of 15.1 years.

**Table 4:** Important factors regarding patient explanation, obtaining consent/assent, and respecting wishes

Items	Counts (% of total)						
	Explanation			Consent		Respecting wishes	
N	55	100	56		100	55	100
In principle	33	58.9	25		44.6	7	12.5
Patient age	13	23.2	28		50.0	6	10.7
Patient’s ability to understand	42	75.0	44		78.6	19	33.9
Contents of the procedure	4	7.1	16		28.6	14	25.0
Guardian’s intention	42	75.0	22		39.3	2	3.6
Not conducted in principle	0	0.0	0		0.0		-
Patient’s emotional stability	20	35.7	15		26.8		-
Joint decision-making process based on the best interests of the patient	-	-	-		-	13	23.2
Physician’s judgment	-	-	-		-	0	0.0
Other	0	0.0	-		-	-	-

#### Assessment of the patient’s understanding

Almost two-fifths (35.7%) of the participants responded that assessment was performed in all cases, half (50%) responded that assessment was performed in some cases, 5.36% responded that assessment was performed in contents, 1.8% responded that assessment was performed at patient’s age, and 7.1% responded that assessment was not performed.

If assessment of patient understanding is conducted, 52 respondents answered the question about who mainly conducted the assessment: 50% were “physicians”, 63.5% were “nurses”, and 17.3% were “professions other than physicians or nurses”. In addition, 19 respondents answered the question about the way of the assessment: 21.1% of them responded “using existing assessment tools” and 79% responded “using original assessment tools”.

### Correlation analysis

Table 5 shows the polychoric correlation coefficients between each of the items regarding clinical difficulties. There was significant positive correlation between item [1] and item [2] (*r* = .809, *p* < .001), which means that when patient’s RMT was an issue, assessment and response to the patient’s RMT was also a problem. There was significant positive correlation between item [2] and item [3] (*r* = .620, *p* = .006) and between item [2] and item [6] (*r* = .620, *p* = .021), which means that when the assessment and response to patient’s treatment was difficult, supporting related patient’s RMT or the assessment of and response to the parent’s RMT were also difficult. There was a significant positive correlation between item [3] and item [4], which means that when the support regarding the patient’s RMT was a problem, IC for the patient was also a problem (*r* = .380, *p* = .046). Item [4] significantly positively correlated with item [5] (*r* = .922, *p* = .017) and item [7] (*r* = .812, *p* = .027), meaning that when explaining the disease and medical treatment for the patient and parent was difficult, obtaining IC for the patient was also difficult.

**Table 5:** Polychoric correlation coefficients between each of the items regarding clinical difficulties

	[1]		[2]		[3]		[4]	[5]		[6]	
[1]											
[2]	0.809^***^										
[3]	0.616		0.620^***^								
[4]	0.372		0.441		0.380^*^						
[5]	0.327		0.474		0.312		0.922^*^				
[6]	0.380		0.376^*^		0.351		0.271	0.347			
[7]	0.208		0.386		0.243		0.812^*^	0.850		0.355	

N = 56 ^*^p < .05, ^**^p < .01, ^***^p < .001

Each items were following: [1] patients’ resistance to and refusal of medical treatment (RMT), [2] assessment of and response to patients’ RMT, [3] support for patients’ RMT, [4] informed consent for patients, [5] explanation of illness and treatment for patients, [6] assessment of and response to parents’ RMT, [7] explanation of illness and treatment for parents.

## Discussion

### Difficulties in obtaining IC for adolescent generation patients

Simple tabulation showed that it is particularly clinically difficult at many hospitals to explain the disease and medical treatment for the patient, to obtain IC for the patient, and to explain disease and medical treatment for parents.

#### Explanation of disease and medical treatment for patient

The implementation of medical explanations to patients is largely consistent with what was reported in previous studies conducted in 2008 [15] and 2013 [16]. However, the contents for which the rate of explanation has been low such as prognosis and fertility are those for which adolescent cancer patients have strong needs [17, 18]. The patients’ information needs may not be being met.

The patient’s wish was under one-fifth of a percent as for the decision on whether or not to provide explanations to patients, and it is suggested that the patient’s own intentions and wishes are not confirmed or are difficult to reflect.

The top reasons for not providing explanations to patients were “giving patients mental anxiety” and “patients not being able to understand”. In this survey, it was inferred that there is a great deal of difficulty in handling very sensitive information such as diseases and treatments, particularly fertility and prognosis, in a way that patients can understand while giving consideration to the mental aspects of adolescent cancer patients.

#### IC for patients

IC was obtained in almost all cases. On the other hand, it was clearly revealed that obtaining consent was not conducted when physicians judged “(because) the patient does not have capacity to make decision.” Furthermore, age of the patient is one of the criteria to judge obtaining consent for patient. However, “patient’s capacity to understand” was the most common and important factor of the explanation, obtaining consent, and respecting for patient will. It suggested that the physician’s judgement for patient’s capacity of decision-making affects providing explanation and obtaining IC for patients.

These results suggest that, in terms of the explanation and IC for patients, those lead to clinical difficulties in cases where there are no clear standards such as the nature of the procedure or at which age of the patient, but also the fact that the physician needs to judge the patient’s developmental age and cognitive developmental levels as the capacity to understand and make decisions.

#### Explanation of disease and treatment for parent

Explanations to parents were related to the issue of IC of patients, and the reality that medical treatment is performed with the consent of parents even without the consent of patients was revealed. These results suggest that the implementation of medical treatment for adolescent cancer patients is largely dependent on the parents’ intentions. Chappuy et al. empirically showed that parents who receive explanations do not always understand the contents of the explanations [19]. It was suggested that it is important to understand the parents who make decisions on behalf of their children in order to provide appropriate treatment, but it is also necessary to assess and care for the psychosocial background, such as medical trauma and family functioning, which can interfere with the parents’ understanding and recognize.

### Factors related to acceptance of the disease by adolescent cancer patients

The results showed that when patient’s resistance and refusal become a problem, assessment and providing support for psychosocial factors behind the resistance and refusal is also difficult. In addition, the results clearly showed that providing support related with patient’s RMT and explanation disease and treatment for patient and parents, obtaining IC for the patient is also difficult. DM support is to assess the psychosocial factors that influence resistance to and refusal of treatment, and to provide support as needed.

In the adult domain, four models of competence related to treatment consent are presented. The four abilities are (1) the ability to express a choice, (2) the ability to understand, (3) the ability to recognize, and (4) the ability to think rationally [20–24]. The MacArthur Competence Assessment Tool series has been developed as a tool to assess these abilities in structured interviews. In a developmental perspective on the capacity to consent, four developmental levels can be estimated: (1) being informed, (2) expressing a view, (3) influencing a decision, and (4) being the main decision-making person [4, 24]. Children’s level of participation in decision-making should be provided by both the child’s own abilities and desires [25, 26]. Moreover, the assessment of children’s capacity to give consent is an unexplored area because it is easily influenced by confounding factors such as the cognitive abilities of the developing children and their relationship with their parents. Leikin points out that DMC is influenced by mechanisms such as illness experience, family situation, family dynamics, and culture, and noted the importance of developmental aspects in children’s and adolescents’ DMC and the need for interventions to promote developmentally appropriate participation [25]. In addition, Hein et al. added the point that not only parents, but also physicians and friends dynamically influence the patient’s will [5].

Especially teenagers are likely to have psychological conflicts and confusion associated with the establishment of ego identity, ambivalence in parent-child separation, and foresight about the future. The acceptance of the disease in this period may affect their mental QOL and independence.

### Informed consent for adolescents with cancer and trauma informed approach

The results of this study suggest that the question is whether informed consent can be given without augmenting adolescents’ anxiety in a clinical setting. In cancer treatment, the risk of medical trauma and PMTS is high due to treatments with strong side effects, treatments with intense pain such as intrathecal injection, changes in appearance and appearance due to chemotherapy, steroids, and surgery, restraints due to prolonged and frequent hospitalization and sterile room management, and repeated highly invasive treatments and tests. Pre-illness traumatic experiences, behavioral and emotional problems, other stress and functional difficulties, and lack of social and psychological support are also risk factors for prolonged stress symptoms and development of trauma. As part of the response to medical trauma, it has been suggested that all health care providers follow the “D-E-F Protocol“[27], and this protocol includes support for the child’s own understanding of what is happening. It is the natural right of children, including adolescents, to be informed about their illness, the treatment and testing they receive, and to participate in the process related to their illness, just as adults are allowed to do. It is important that these rights are protected in preventing their trauma. However, a child’s wishes may be greatly influenced by family relationships and relationships with medical staff, and may also reflect the child’s own developmental and personality characteristics. Adolescents in particular are sensitive to the evaluations of others, are prone to ambivalent feelings between independence and dependence, and tend to make risky choices. It will be necessary to understand the patient’s expressed intentions and wishes comprehensively from a biopsychosocial perspective and support them with a trauma-informed approach.

### Assessment required to support patient decision-making

The results suggested that assessment and support for child’s and adolescents’ refusal of treatment were identified as issues to be addressed, but only half of the patients were assessed for their understanding related with their disease, and each facility relied on its own original method. It is necessary to develop an assessment tool for disease acceptance of adolescent that provides certain standards and establish it for use in the field so that it can be applied in the field.

Four-factor model of decision-making capacity [4, 24] may be useful in assessing disease acceptance. Koelch et al. developed the MacArthur Competence Assessment Tool for Clinical Research (MacCAT-CR) based on the four-factor model of decision-making, to examine the practicality of the MacCAT-CR for children and their understanding of and ability to perform the disclosure component of a clinical trial [28]. The subjects were 12 children between the ages of 7 and 12 with a diagnosis of attention deficit hyperactivity disorder (ADHD) or oppositional defiant disorder (ODD) and their parents. Results showed that the MacCAT-CR could be administered to the children. Children’s understanding was lower than that of their parents. In addition, clinicians assessed all affected children as competent, but all were judged to be incompetent in the MacCAT-CR. Koelch et al. described the following [28]. There is a need to validate using external indicators as to whether ability is being assessed effectively, and that the difference between the scores obtained from the clinician’s assessment and the MacCAT-CR suggests that children may be convinced even though their understanding is not complete. Hein et al. examined the assessment of capacity to consent in 17 outpatient children and adolescents (aged 6–18 years) before undertaking genetic tests [29]. The results showed that children may have capacity to consent at 11.8 years of age.

These studies demonstrated the practical feasibility of using the MacCAT scale with pediatric populations. In addition, Hein et al. made the following modifications to the MacCAT-CR in making it a pediatric and adolescent version, checking for reliability and validity [5, 30]. It was recommended that visual cards be used in combination and they added sample statements of interventions and questions in order to examine systematic effects. The two questions were scored. Finally, results showed high reproducibility of the MacCAT-CR total and subscale scores. Age was a good predictive factor of MacCAT-CR ability: children younger than 9.6 years had low ability (sensitivity 90%) and children older than 11.2 years had high ability (specificity 90%). The optimal cut-off age was 10.4 years (sensitivity 81%, specificity 84%).

As other research has highlighted, the assessment of disease acceptance for adolescent cancer patients should be based on the consequences in their daily life ( as one of the factors that shape the self) and social relationships (for example family and friends). Along with assessment, the practice of trauma-informed care will lead to the establishment of further DM. Through this assessment, if there are issues in emotional functioning, family functioning, cognitive functioning, and so forth, decision-making support by appropriate specialists should be provided for these situations.

## Limitations

This study was based on a questionnaire from pediatric oncologists at a pediatric cancer base hospital, and we need to be cautious about transferring the results to dealing with adolescent patients who are treated by orthopedic surgeons, neurosurgeons, and hematologists in the medical field with adults. If pediatricians consider explaining medical conditions to the adolescent generation as a continuation of the explanations and consent obtaining procedures they provide to prepubescent children, there may be a greater chance that IC will be inadequate. In addition, the number of responses from the targeted physicians was small, and we did not examine the effects of differences in years of experience in pediatric cancer care, physicians’ gender and age. This survey was an indirect request, and the importance of this survey may not have been fully conveyed to physicians who are busy with their clinical work. In this sense, more physicians interested in this topic may have responded to the survey. A separate survey is needed to identify the psychosocial issues of actual patients and their families.

## Conclusions

There are still issues in the assessment of the acceptance of illness and adolescent’s decision-making capacity based on psychosocial factors of patients, and there is difficulty in support for decision-making. Therefore, there is a need for development of an assessment tool for patient’s decision-making capacity and acceptance of illness considering the unique adolescent mentality.

## Electronic supplementary material

Below is the link to the electronic supplementary material.

Additional File: Questionnaire on disease acceptance and decision-making among AYA cancer patients

## Data Availability

The datasets analyzed during the current study are not publicly available due to be planed a developmental analysis but are available from the corresponding author on reasonable request.

## List of Abbreviations

IC: informed consent
DMC: decision-making capacity
DM: decision-making
RMT: refusal of medical treatment
PMTS: pediatric medical traumatic stress
