# Supplementary material for: Medical staff’s sense of awareness of informed consent for adolescent cancer patients and the need for decision-making support practiced from the perspective of trauma-informed approach
Source: BMC Med Ethics. 2023 May 6;24:28. doi: 10.1186/s12910-023-00907-y (PMC10164311; doi:10.1186/s12910-023-00907-y)
Supplement: Supplementary file 1 — Additional File: Questionnaire on disease acceptance and decision-making among AYA cancer patients [file 12910_2023_907_MOESM1_ESM.docx]

**Questionnaire on disease acceptance and decision-making among AYA cancer patients**

Although there is a wide range of definitions of the AYA generation, both narrowly and broadly defined, for the purpose of this study, we will refer to pediatric and AYA generation cancer patients **between the** ages of **12 and 20.** We would appreciate your cooperation in the following.

**If you are willing to cooperate, please tick the box below to confirm your agreement.**

**□ I agree to cooperate with this study**

1. **What are some of the issues in clinical practice regarding explanation and consent for AYA cancer patients ("patients")?**

(1) Patient resistance/refusal to treatment

　　Often, Sometimes, Neither, Rarely, Never

(2) Assessment of and response to patient resistance/refusal to treatment

　Often, Sometimes, Neither, Rarely, Never

(3) Assistance regarding patient resistance or refusal of treatment

　Often, Sometimes, Neither, Rarely, Never

(4) Informed consent for patients

　Often, Sometimes, Neither, Rarely, Never

(5) Explanation of disease and treatment to patients

　Often, Sometimes, Neither, Rarely, Never

(6) Assessment and response to parental refusal of treatment

　Often, Sometimes, Neither, Rarely, Never

(7) Explanation of illness and treatment to parents

　Often, Sometimes, Neither, Rarely, Never

(8) Others

( )

**2. we would like to ask you about the explanations you give to the patients themselves when you provide medical care.**

**Please tick the boxes that apply to you.**

(1) Do you provide explanations to patients regarding medical treatment?

☐ Perform in all cases

　　　　☐ Varies from case to case

　　　　☐ Varies depending on content

　　　　☐ No ☐ No

(2) At what age would you start explaining to patients?

Approximately ( ) years old or older

□ No specific age

□ In principle, not explained to patients.

(3) Would you explain the following to the patient?

In principle, please select all that you would like to explain.

　　　　☐ Name of disease or diagnosis

　　　　☐ Disease status/pathology

　　　　☐ Treatment and its methods

☐ Treatment and its significance

　　　　☐ Treatment and its side effects

　　　　☐ Late complications

　　　　☐ Fertility

　　　　☐ Schooling and employment

　　　　☐ About the support you can get

　　　　Other ( )

(4) Would you explain the following conditions to a patient?

　　 In principle, please select all that you would like to explain.

□ Relatively mild disease

□ A medical condition that is not immediately life-threatening, but requires continuous treatment, may cause permanent injury, or is not considered a minor illness.

□ If the patient has a serious, life-threatening medical condition

When the patient is not expected to recover from the treatment and is judged to have a short life expectancy.

(5) What factors are important in deciding whether or not to explain to the patient?

　　 Please select up to 3 that best apply.

□ Explained in principle.

□ Decided mainly based on patient age

□ Decided mainly based on patient's ability to understand

☐ Decisions are made primarily with the patient's emotional stability in mind.

□ Decided mainly based on diseases and procedures

□ Decisions are made mainly in consideration of the wishes of parents and other guardians.

□ In principle, not explained to patients.

Other ( )

(6) How do you decide whether or not to explain to the patient?

　　　　　　☐ In accordance with the rules of the hospital

☐ At the discretion of the physician

　　　　　　☐ At the discretion of the multidisciplinary team

　　　　　　☐ At the discretion of the parent

　　　　　　☐ At the patient's request

(7) If you do not explain to the patient, for what reasons do you not explain?

　 Please select all that apply.

□ Because the patient does not understand

□ Because it causes mental anxiety to the patient.

□ I don't have enough time to explain.

□ Because explanation to parents and other guardians is sufficient.

□ Because explanations to patients are left to parents and other guardians.

□ Because I don't know how to explain to the patient or it is difficult to explain to the patient

□ Because it is not reflected in reimbursement

Other ( )

(8) Who is primarily responsible for explaining to patients? Please select the one that best fits your question.

□ Mainly physicians

□ Mainly nurses

□ Mainly other professions other than physicians and nurses

(Job title: )

☐ Case by case

If so, what type of work will be done? Please select all that apply.

☐Doctor ☐Nurse ☐Other ( )

(9) How long does it take you to provide explanations to patients?

□ 0-5 min.

6-10 minutes

10-20 min.

20-30 min.

More than 30 min.

(10) What methods do you use to explain to patients? Please select all the methods you use.

□ Explain verbally

□ Explain using pictures and charts

□ Research and training opportunities

□ Specialized staff is in place.

Other

　　→Please describe the details of your initiatives.

| Other specific initiatives |
| --- |

**3. We will ask about your hospital's policy on obtaining "consent" (*) from the patients themselves for medical treatment.**

*In this questionnaire, "consent" is defined as taking informed consent when the individual has the capacity to consent.

(1) Do you obtain the patient's own consent for medical treatment?

□ May obtain (□ Sign document □ Verbally only )

No

(2) At what age do you obtain the patient's own "consent"?

Approximately ( ) years old or older

□ No specific age is set.

□ In principle, "consent" is not obtained from the patient.

(3) Do you obtain the patient's own "consent" for the following procedures?

　 In principle, please select all that you "agree" to.

□ Blood sampling □ Simple X-ray □ MRI

□ Lumbar spine examination and bone marrow examination □ Cardiac catheterization □ Endoscopy

□ Biopsy (kidney, liver) □ Electroencephalography, electrocardiogram □ Surgical operation

□ Medications □ Life restrictions (nutrition, diet, behavioral restrictions, etc.)

□ Chemotherapy □ Radiotherapy □ Medication not covered by insurance

(4) What factors are important in deciding whether to obtain the patient's own "consent"?

Please select the three most applicable.

□ In principle, the patient's own "consent" is obtained.

□ Decided mainly based on patient age

□ Decided mainly based on patient's ability to understand

☐ Decisions are made primarily with the patient's emotional stability in mind.

□ Decided mainly based on diseases and procedures

□ Decisions are made mainly in consideration of the wishes of parents and other guardians.

□ In principle, the patient's own "consent" is not obtained.

(5) For what reasons do you not obtain the patient's own "consent"? Please select the one that best applies.

□ Because the patient does not have the ability to make a "consent" decision.

□ Because it is sufficient to obtain "consent" from parents or other guardians.

□ If patients say they don't want to do it, it is difficult to perform medical treatment.

Other ( )

(6) Even if you obtain "consent" from the patient himself/herself, do you obtain "consent" from parents or other guardians?

□ In principle, "consent" is also obtained from parents and other guardians.

□ In principle, "consent" is not obtained from parents or other guardians.

(7) If the patient does not "consent" but a parent or other guardian gives consent, would you perform the medical procedure in question? Please choose one that comes closest. (Please answer this question assuming that the patient does not give his/her consent even after you have tried to obtain his/her "consent.)

□ Not implemented in principle.

□ In principle, it will be implemented.

Can't say either

Other ( )

(8) If you have obtained the "consent" of the patient, but the parent or other guardian refuses, would you perform the relevant medical procedure? Please choose one that is closest to your answer. (Please choose one that is closest to your answer.) (Please answer this question if you have tried to obtain the consent of the parent or guardian but were unable to do so.

□ Not implemented in principle.

□ In principle, it will be implemented.

Can't say either

Other ( )

**4 We ask you about obtaining assent* from the patients themselves when providing medical care to them.**

*In this questionnaire, "assent" is defined as obtaining informed consent by providing an easy-to-understand explanation to the extent that the individual is able to understand, in cases where the individual does not have the capacity to consent. Assent is also called "understanding" or "assent.

(1) Do you obtain the patient's own assent, even if you do not obtain the patient's own "consent"?

□ May obtain (□ Sign document □ Verbally only)

No

(2) At what age do you obtain the patient's own assent?

Approximately ( ) years old or older

□ In principle, no assent is obtained from the patient.

(3) Do you obtain the patient's own assent for the following procedures? In principle, please select all that obtain assent.

□ Blood sampling □ Simple X-ray □ MRI

□ Lumbar spine examination and bone marrow examination □ Cardiac catheterization □ Endoscopy

□ Biopsy (kidney, liver) □ Electroencephalography, electrocardiogram □ Surgery

□ Medications □ Life restrictions (nutrition, diet, behavioral restrictions, etc.)

Chemotherapy □ Radiotherapy

□ Medication not covered by insurance

(4) What are the most important factors in deciding whether to obtain the patient's own assent? Please select one that most applies.

□ As a rule, we try to obtain the patient's own assent.

□ Decided mainly based on patient age

□ Decided mainly based on patient's ability to understand

□ Decided mainly based on diseases and procedures

□ Decisions are made mainly in consideration of the wishes of parents and other guardians.

□ In principle, the patient's own assent is not obtained.

(5) For what reasons do you not obtain the patient's own assent? Please choose one that best applies.

□ Because the patient does not have the ability to make an assent decision.

□ Because it is sufficient to obtain "consent" from parents or other guardians.

□ If patients say they don't want to do it, it is difficult to perform medical treatment.

Other ( )

**5. ask about the meaning of obtaining the patient's "consent" or assent.**

(1) For what reason or effect do you obtain "consent" or assent from the patients themselves? Please select all that apply.

□ Because the patients themselves have the right to make their own decisions.

□ (Even when "consent" cannot be obtained from the patient), the patient's own wishes and opinions should be respected.

□ To relieve patient anxiety

□ To get patients to cooperate

□ It is the patient's right to obtain "consent" or assent from the patient himself/herself.

□ No "consent" or assent is obtained from the patients themselves.

Other ( )

**6. if a parent or guardian has given consent for a medical procedure and the patient himself/herself refuses, are there any cases in which the medical procedure is not performed in accordance with the patient's own wishes?**

(1) At what age do you follow the patient's own refusal?

Approximately ( ) years old or older

☐ Depends on the nature of the refusal

　　　　　　(Specify: )

□ In principle, the patient's own refusal is not followed.

(2) What factors do you consider important in deciding whether to comply with the patient's own wishes to refuse a medical procedure? Please select one that most applies.

□ In principle, the patient's own wishes are followed.

□ Decided mainly based on patient age

□ Decided mainly based on patient's ability to understand

□ Decisions are made primarily based on the disease, the nature of the procedure, and the prognosis if the procedure is not performed.

□ In principle, medical treatment is performed based on the consent of a parent or other guardian, even if the patient himself/herself wishes to refuse.

□ Collaborative decision-making process based on the best interests of the patient

□ Your doctor is making the decision.

**7. ask about the reaction of the patient, parents, or other guardians to the refusal.**

(1) What do you think is associated with the patient's own intention to refuse medical treatment?

　　　　　　　☐ Physical pain and distress from treatment

　　　　　　　☐ Emotional instability

　　　　　　　☐ Parents' or guardians' intention

　　　　　　　☐ Not being informed about the treatment or procedure

　　　　　　　☐ Not being informed about the disease

　　　　　　　Other ( )

(2) What do you think is associated with parents' or other guardians' willingness to refuse medical treatment?

☐ Physical pain and distress of the child due to treatment

☐ Child's emotional instability

☐ Emotional instability of parents

☐ Child's intention

☐ Lack of knowledge or lack of understanding about parent's treatment or procedure

☐ Child is not informed about the disease

Other ( )

(3) If a parent or other guardian refuses to give "consent" or assent to a patient, what do you think is behind this refusal?

☐ Child's emotional instability

☐ Emotional instability of parents

☐ Child's intention

☐ Lack of knowledge or lack of understanding about parent's treatment or procedure

☐ Child is not informed about the disease

Other ( )

**8. if "consent" or assent is obtained, we would like to ask about the subsequent reactions of the patient, parents or other guardians, and how they responded.**

1. Do you conduct an assessment of the patient's understanding?

☐ Perform in all cases

☐ Will be done in some cases

☐ Will be done depending on the content.

　　(Example: )

☐ Do depending on age

　　　Approximately ( ) years old or older

☐ No ☐ No

1. If you are conducting an assessment of the patient's understanding, who is doing it?

☐ Mainly physicians

☐ Mainly nurses

☐ Mainly other professions other than physicians and nurses

(Job Title)

1. If you are conducting an assessment of your understanding of the patient, how is this done?

　　　　　　　☐ Use existing assessment tools

　　　　　　　　　(Tool name: )

☐ Use original assessment tools at the facility

　　→Please be specific about the contents.

Specifics

**9. please feel free to describe any opinions your hospital has about explaining to the patient, "consenting", obtaining assent, etc.**

Thank you for your cooperation.
